# Application of integrated nested Laplace approximation to identify hot spots of methylation heterogeneity in healthy individuals from the MAMELI cohort

**DOI:** 10.3389/fgene.2026.1787544

**Published:** 2026-04-29

**Authors:** Tiago Nardi, Eva Dariol, Rachele Matsagani, Donya Zojaji, Stefano Gustincich, Luca Pandolfini, Elia Biganzoli, Valentina Bollati

**Affiliations:** 1 ‘LETE’ Laboratory of Environmental and Translational Epigenetics, Department of Biomedical and Clinical Sciences, University of Milan, Milan, Italy; 2 Non-Coding RNAs and RNA-Based Therapeutics, Istituto Italiano di Tecnologia (IIT), Genova, Italy

**Keywords:** DNA methylation (5mC), epigenome-wide association studies, exposome, generalized linear mixed models, integrated nested Laplace approximation, methylation regulation, methylation variance, nanopore sequencing

## Abstract

DNA methylation is an epigenetic regulator of gene expression and cell identity, which can be shaped by both physiological and pathological factors, including environmental exposure. The identification of sites with high methylation variability can be computationally challenging, especially in large-scale studies. To address this, we propose a framework based on the integrated nested Laplace approximation (INLA) to model methylation with Bayesian generalized linear mixed models (GLMMs), accounting for subject covariates, genomic annotations, and cell composition. To validate the methodology, we sequenced 158 healthy subjects with nanopore and analyzed a panel of 13 genes related to inflammation and stress response. We identified a set of hypervariable CpG sites whose genomic context and methylation levels were consistent with a regulatory role, making them potential candidates for epigenomic association studies. In our comparison, INLA results were concordant with those obtained with MCMC-based methods, with runtimes shorter by orders of magnitude. The computational efficiency of the framework allows for fast exploratory data analysis, model testing, and iterative prototyping, making it viable for large-scale studies that otherwise would be computationally prohibitive.

## 1 Introduction

Cell development and function depend on epigenetic regulation, with DNA methylation among the most studied mechanisms (Lim and Maher, 2010). The predominant form in mammals is 5-methylcytosine (5mC), which accounts for 2%–5% of all cytosines (Moore et al., 2013).

Variations in DNA methylation have been linked with many biological processes, both physiological and pathological, including aging, inflammation, and environmental factors (Horvath, 2013). Epigenome-wide association studies (EWASs) characterize these profiles, associating specific methylation signatures with exposures. To reduce costs, it has been proposed to focus on a subset of sites, namely, CpG sites with high inter-individual methylation variability (Hachiya et al., 2017). Consistently, genomic regions with variable sites are enriched for regulatory elements, including transcription factor binding sites and chromatin states such as enhancers and active transcription start sites (Gu et al., 2016).

Despite this, few studies have directly analyzed epigenome-wide patterns of DNA methylation variance (Kiltschewskij et al., 2025). It is reflected in the available methods, many of which focus on finding methylation differences between groups (Dolzhenko and Smith, 2014; Halla-aho and Lähdesmäki, 2020) rather than variations within groups. Furthermore, various methods designed to detect variations have a limited capability to handle subject covariates (Saddiki et al., 2022).

A valid solution would be to model the variability with Bayesian generalized linear mixed models (GLMMs), which can include subject covariates and sequence features such as CpG islands, while accounting for nested relationships. Furthermore, Bayesian GLMMs provide robustness to unbalanced data or sparse observations, common in many datasets, by enabling uncertainty quantification and partial pooling (McElreath, 2020). However, implementations based on MCMC, including Hamiltonian Monte Carlo (HMC), are often computationally intensive. As EWAS datasets can include hundreds to thousands of subjects, these methods can become computationally intractable (Bakulski et al., 2023).

Approximate Bayesian methods have increasingly been used as faster alternatives (Li et al., 2025). One of them is the integrated nested Laplace approximation (INLA), which is based on latent Gaussian models (Rue et al., 2009). In this study, we used INLA to identify genomic sites with high inter-individual methylation variability and compared its performance with brms, an MCMC-based method. For testing the methods, we sequenced a healthy cohort using nanopore, analyzing a panel of inflammation- and stress-related genes.

## 2 Methods

### 2.1 Subjects

We analyzed 158 healthy blood donors who were recruited from AVIS Legnano (Associazione Volontari Italiani Sangue) as part of the first 200 healthy participants enrolled in the MAMELI cohort (total cohort size: n = 6,200). Detailed recruitment procedures and eligibility criteria, including the ethics committee approval, have been previously described (Bollati et al., 2025). In brief, the eligible participants were adult blood donors (aged ≥18 years) residing in the city of Legnano. Before enrollment, we received written informed consent from all participants. We excluded individuals with chronic diseases, including cardiovascular diseases, a cancer diagnosis within the previous 2 years, or any condition impairing the ability to provide informed consent.

### 2.2 Samples and library preparation

We collected peripheral blood samples (7 mL) in EDTA tubes that were then transported to the University of Milan. At the time of collection, a complete blood count with differential was performed for each participant. Due to logistical reasons, blood counts were unavailable for two participants. We centrifuged samples at 1,300 x g for 15 min at room temperature to isolate the buffy coat within 4 h of phlebotomy.

Genomic DNA was extracted using the Gentra® Puregene® Blood Kit (QIAGEN, Hilden, Germany, 158026) according to the manufacturer’s instructions. DNA concentration was measured using the QuantiFluor® dsDNA System (Promega Corporation, Madison, WI, United States, E2670), and purity was assessed using a NanoDrop spectrophotometer (Thermo Fisher Scientific, Waltham, MA, United States).

We sheared the DNA to a target fragment length of 15 kb using Covaris g-tubes (Covaris, Woburn, MA, United States, 520079) with centrifugation at 2,100 x g for 60 s. After shearing, we used the Genomic DNA ScreenTape Assay (Agilent, Santa Clara, United States) to evaluate the fragment size distribution.

We prepared sequencing libraries using the Ligation Sequencing Kit V14 (Oxford Nanopore Technologies) following the manufacturer’s protocol. For each sample, we loaded 1,000 ng of DNA onto PromethION Flow Cells (Oxford Nanopore Technologies) for sequencing.

Full-blood counts and differentials were obtained by AVIS from each participant at collection.

### 2.3 Sequencing and basecalling

For basecalling and modification calling, we used Dorado v.0.8.2 with the sup model v 5.0.0 for the detection of 5mC and 5-hydroxymethylcytosine (5hmC) in CpG dinucleotides (5mCG_5hmCG). We aligned reads to the T2T-CHM13v2.0 human genome reference using minimap2 v. 2-2.28 (r1209). We used modkit v. v0.3.1 with the default parameters to extract and summarize the results as a bedMethyl, retaining 5mC calls for downstream analyses.

### 2.4 Statistical analysis

We conducted all statistical analyses in R (version 4.5.2) using the Tidyverse package collection (Wickham et al., 2019) and Bioconductor (Huber et al., 2015). A complete list of R packages and their corresponding versions (“sessionInfo.txt”) is available in the publication-associated Dataverse.

#### 2.4.1 Dataset

We formed a panel of genes that are sensitive to environmental stimuli, selecting 13 genes involved in inflammation, stress response, and metabolism. For inflammation, we included *IL-6* (Hirano, 2021), *CRP* (Sproston and Ashworth, 2018), *IFNA1* (Jeon et al., 2023), and *NFKB1* (Liu et al., 2017). For stress response, we selected *NR3C1* (Palma-Gudiel et al., 2015), *FKBP5* (Zannas et al., 2016), *CRH* (Webster et al., 1998), *POMC* (Slominski et al., 2000), *CYP21A2* (Nordenström et al., 2017), *BDNF* (Notaras and van den Buuse, 2020), *SLC6A4* (Stoffel et al., 2023), and *OXTR* (Szeto et al., 2017). Then, we included *FTO* for its role in regulating metabolism and inflammation (Li et al., 2024).

We selected CpG sites within these 13 genes, including promoter regions, defined as the 2,000 base pairs upstream of the transcription start site.

We retained the 100 samples with the highest mean sequencing coverage, including sites with a minimum coverage of 10 reads in at least 90 samples.

#### 2.4.2 Dataset filtering

To reduce computational overhead, we filtered sites with limited methylation variability. We used normalized Shannon entropy (Hrel) (Wilcox, 1973) to prioritize distributional evenness across subjects rather than the absolute magnitude of differences. We calculated Hrel using 10 bins, corresponding to the minimum coverage (10×), to measure variation at the minimal resolution.

To validate the measure, we compared Hrel with the interquartile range (IQR) and standard deviation (SD) using pairwise Spearman rank correlation on the full dataset. Then, we filtered at incremental 10% percentile thresholds across all three metrics and calculated the Jaccard similarity for each dataset pair.

We fitted the complete model to nine datasets, filtered at the 40th, 50th, and 60th percentiles of each metric, to assess the sensitivity of the results to the different thresholds. We compared the obtained site × subject interaction effects across models using Pearson and Spearman correlations on the shared genomic sites.

#### 2.4.3 Model building and comparison

We fitted Bayesian GLMMs using the INLA framework using the package R-INLA v. 25.06.07 (Rue et al., 2009). To model the number of methylated and unmethylated reads at each genomic site, we specified a binomial likelihood.

We fitted a set of candidate models, including different combinations of population-level (fixed) and group-level (random) effects, using the dataset filtered at the 50th Hrel percentile. We selected models on the basis of the deviance information criterion (DIC) (Roos and Held, 2011), the Watanabe–Akaike information criterion (WAIC) (Watanabe, 2010), and the conditional predictive ordinate (CPO) (Pettit, 1990). In cases of comparable model fit (score differences under four), we preferred the model with the lowest number of effective parameters.

The tested population-level effects included annotation (coding sequence, untranslated region, intron, and promoter region) and the presence of CpG islands or transposable elements. The group-level effects included the subject, gene, transposable element (TE) taxonomy, genomic site, and site × subject interaction.

All group-level effects were included as independent and identically distributed (iid) random effects. We preferred modeling genomic sites as iid rather than using spatial correlation (e.g., random walk) because our aim was to identify site-specific variation, and spatial smoothing would shrink the differences between adjacent sites.

#### 2.4.4 Prior specification and sensitivity analysis

We tested the prior sensitivity as each term was added, comparing posterior means and CIs. The selected priors were kept in the subsequent models.

For population-level effects, we used a normal prior with a mean of 0, testing standard deviations of 0.5, 1, 1.5, 2, 3, 5, and 10.

For group-level effects, we specified penalized complexity (PC) priors (Simpson et al., 2017) for the standard deviation using the tail probability formulation *P*(σ > u) = α. We tested u values from 0.5 to 2 and α values of 0.01 and 0.05.

#### 2.4.5 Complete model

In the complete model, we assigned a normal (0, 3) prior to the intercept. The population-level effects included annotation and TE presence, using Normal(0, 2) and Normal(0, 1.5) as the priors.

Group-level effects were specified as iid effects and included the subject [*P*(σ > 1) = 0.01], TE taxonomy [*P*(σ > 1) = 0.05], genomic site [*P*(σ > 1.5) = 0.05], and site × subject interaction [*P*(σ > 1) = 0.05]. 

To assess computational scalability, we fitted the model with increasing subsets of genes (3, 5, 7, 10, and 13). We recorded the runtime and the memory footprint of the fitted model object as a proxy for RAM requirements using the R package lobstr v. 1.2.0 (Wickham, 2026).

#### 2.4.6 Comparison to brms

To validate the INLA approach, we fitted the complete model with brms v. 2.23.0 (Bürkner, 2017), which is based on MCMC sampling.

To reduce computational overhead, we used the dataset filtered at the 60th Hrel percentile.

For population-level effects, we specified the same priors used for INLA.

For group-level effects, we specified equivalent exponential priors on the standard deviation. In summary, matching the exponential tail probability *P*(σ > u) = exp(−λu) with the PC prior definition yielded the rate parameter λ = −ln(α)/u.

We ran four chains with four threads each for 2,000 warmup iterations, followed by 1,800 sampling iterations, and assessed the convergence through effective sample size (ESS) and R̂ values.

To measure the concordance between brms and INLA, first, we compared the population-level effects and variance components. Then, we measured the Pearson and Spearman correlation for each site × subject interaction effect and their site-level summaries (mean, IQR, SD, and range).

#### 2.4.7 Cell-type composition adjustment

To assess the effects of cell-type composition on methylation, we included the blood cell composition data in the complete model and compared the predictions.

To account for the compositional data structure (percentage of lymphocytes, monocytes, eosinophils, basophils, and neutrophils), we applied a centered log-ratio (CLR) transformation. Then, we excluded neutrophils, which were the most abundant and least variable cell type, and standardized the values to z-scores.

We included the transformed proportions as varying slopes across genomic sites, using PC priors on the standard deviation. For the prior sensitivity analysis, we tested four scale parameters (0.01, 0.05, 0.1, and 0.3), selecting *P*(σ > 0.1) = 0.05 for the final model.

Eosinophil and basophil counts were excluded from the final model as unidentifiable due to their prior sensitivity and wide posterior credible intervals (CI).

To prevent the identification of the two subjects with missing data, we imputed their values by drawing from a normal distribution centered at 0 with an SD of 0.1. To ensure that imputation was not affecting the results, we fitted the model by replacing the imputed values with NA and compared the results.

To quantify how cell composition affected the inter-individual methylation variability, we measured the Pearson and Spearman correlations for site × subject interactions and the site-level summaries of the interactions (IQR and SD of the posterior means across subjects) between the model with and without cell compositions.

We considered a genomic site specifically affected by a cell type when the 95% credible interval (CI) for its corresponding varying slope excluded 0.

#### 2.4.8 Identification of hypervariable sites

To identify CpG sites exhibiting high inter-individual methylation variability, we extracted the posterior means of the site × subject interaction effects from the cell composition-adjusted model. For each genomic site, we computed the IQR of these interaction effects across subjects and ranked the sites accordingly, with higher IQR values indicating greater inter-individual methylation variability.

Genomic sites with variable methylation are expected to be enriched in chromatin states associated with regulation (e.g., enhancers) and depleted in quiescence-associated states (Gu et al., 2016). Therefore, a plausible selection of hypervariable sites should show a distinct pattern in chromatin states compared to the genomic background. Taking this into account, we tested chromatin state enrichment across a range of IQR thresholds (0.10–0.35, in increments of 0.01). For each threshold, we intersected the genomic coordinates of the selected sites with chromatin state annotations from the Roadmap Epigenomics Project using the E030 sample (“primary neutrophils from peripheral blood”) (Roadmap et al., 2015). To lift the coordinates to the T2T-CHM13v2.0 reference, we used rtracklayer v1.68.0 (Lawrence et al., 2009) with the hg38ToHs1 chain file (UCSC Genome Browser) on the E030 annotations pre-lifted to hg38. We mapped 396,835 intervals out of 397,167, retaining the longest fragment when a single interval had multiple mappings.

We modeled enrichment by fitting a Poisson regression in INLA, with the number of hypervariable sites in each chromatin state as the response and expected counts, which were proportional to each state’s frequency in the full dataset, as an offset. We considered a chromatin state to be enriched or depleted when its 95% credible interval for the log enrichment ratio did not include 0. We selected the IQR threshold at which the chromatin state patterns stabilized. To assess the stability of the results, we repeated the enrichment analysis using the effects from all nine filtered datasets and the brms model, testing IQR thresholds of 0.15, 0.20, and 0.25.

## 3 Results

### 3.1 Dataset filtering

The initial dataset had 9,573 CpG sites with coverage higher than 10×.

**FIGURE 1:**
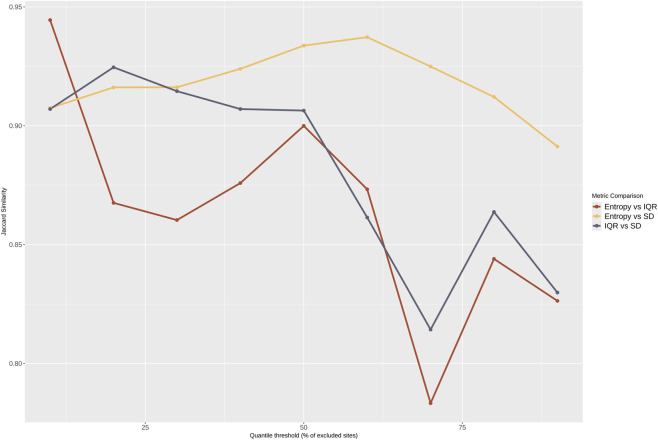
Concordance in the filtered sites using Hrel, IQR, and SD metrics with increasing thresholds of percentile distribution. The X-axis shows the threshold percentiles. The Y-axis shows the Jaccard similarity of pairwise comparisons starting from 0.75.

The three filtering metrics were highly correlated (Spearman’s ρ between 0.95 and 0.98). The agreement in site selection was high, with a Jaccard index higher than 0.78 across all thresholds (Figure 1). Hrel and SD remained highly concordant, with Jaccard similarity consistently remaining above 0.90. Agreement between IQR and the two metrics declined at higher thresholds, reaching a minimum at the 70th percentile.

### 3.2 Prior sensitivity

For all population-level effects, the posteriors were insensitive to the choice of prior.

For group-level effects, the posteriors were stable for the subject, site, and site × subject interactions. The posteriors for TE taxonomy showed sensitivity to PC prior specification (Supplementary Figures 1, 2), especially for more restrictive priors [i.e., *P*(σ > 0.5) = 0.01]. The posteriors converged under less informative priors (i. e., u ≥ 1), indicating sufficient information for variance estimation. We set the prior specifying *P*(s > 1).

### 3.3 Model fitting

We selected the model that balanced the complexity and goodness of fit based on DIC, WAIC, and CPO, which were concordant. Inclusion of the genomic site as a group-level effect captured information from other overlapping site-specific predictors, such as CpG island annotation and gene identity. When we included these predictors together, we observed negligible increases in model fit, approximately two units in the DIC, WAIC, and CPO scores. For this reason, in the final model, we excluded the predictor gene and CpG islands.

The tables with the DIC, WAIC, and CPO scores are available in the publication-associated Dataverse.

### 3.4 Robustness to the filtering criteria

Site estimates of methylation variability between individuals were stable across all filtering thresholds; pairwise comparison of the interaction effects on shared sites had Spearman and Pearson correlations above 0.99 for both IQR and SD of the posterior means.

Given the low sensitivity to filtering choices for downstream analyses, we used the dataset filtered at the 50th percentile of Hrel. The exception was the brms comparison, for which we used the 60th Hrel percentile dataset to reduce computational burden.

### 3.5 Comparison to brms

The brms model showed partial convergence. The main limitation regarded the variance component of the transposable element taxonomy (ESS 80, $\hat{R}$ = 1.08), which was the only parameter with divergent estimates (brms: 0.201, INLA: 0.0515). Genomic annotations and sites had low ESS (81-171) but acceptable $\hat{R}$ , which were 1.04 for the annotations and 1.01 for the sites. The site × subject interaction had the highest convergence ( $\hat{R}$ = 1, ESS >2,000).

Aside from the TE taxonomy, the posterior means and variances between INLA and brms were concordant, including the site (brms: 1.62, INLA: 1.63) and the interaction effect (brms: 0.330, INLA: 0.323).

**FIGURE 2:**
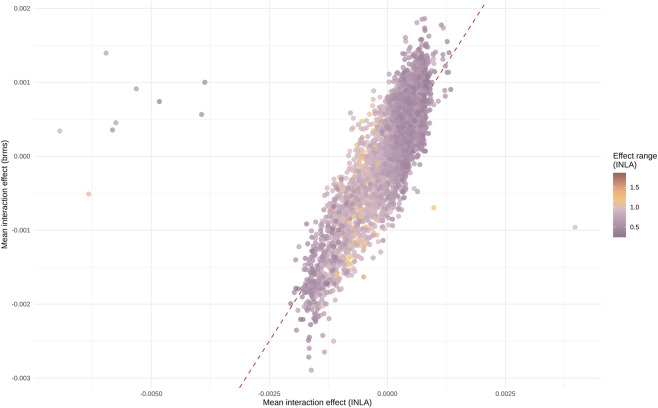
Comparison of INLA/brms estimated mean interaction effects (site × subject) using the dataset filtered at 60th percentile of Hrel. Each dot is a genomic site. The X-axis shows the mean of the posterior interaction effects estimated with INLA. The Y-axis shows the mean of the posterior interaction effects estimated with brms (Spearman ρ = 0.83). Dots are colored according to the estimated mean effect range with INLA, i.e., the maximum mean posterior effect minus the minimum mean effect for the site.

The site × subject interactions had almost complete correlation (Pearson r > 0.99). Site-level summaries for interactions were similarly concordant in variability measures, with Pearson r > 0.99 for IQR, SD, and range. Instead, the site-level mean showed lower concordance (Pearson r = 0.79, Spearman ρ = 0.83), driven by a small subset of sites (Figure 2). This difference did not affect site selection as it was performed using the IQRs.

### 3.6 Computational burden

Models fitted without the interaction term showed reduced computational time, approximately 1 min. Fitting the complete model took approximately 6 min with INLA and 122 h with brms, with a mean chain execution time of 120 h. The cell composition-adjusted model took 14 min.

Memory usage and runtime scaled approximately linearly with the number of observations (sites × subjects) across the tested range. With 208,406 observations, model fitting required 1.8 min and occupied 1.9 GB of RAM. With 476,465 observations, the runtime increased to 5.7 min, and memory consumption increased to 4.2 GB.

The runtimes for each model are available in the publication-associated Dataverse.

### 3.7 Cell-type composition adjustment

The posterior standard deviations for lymphocytes and monocytes were stable across all priors (0.081 and 0.037). In contrast, precision estimates for eosinophils and basophils ranged by orders of magnitude (e.g., with the most restrictive prior for basophils, the 95% CI was 38,000–13,800,000) and were removed from the final model as not identifiable from the data. We tested the inclusion of the population-level effects for cell counts, with no (WAIC) or limited (ΔDIC <3) benefits, and they were not retained in the final model.

The imputation for missing subjects did not affect the estimates, which were almost identical, i.e., Pearson r > 0.99 for both lymphocyte and monocyte slopes, with a maximum absolute difference <0.004 on the logit scale.

A total of 350 sites had lymphocyte-specific effects, and nine sites had monocytes-specific effects.

The models with and without cell-type adjustment showed high concordance (r = 0.99 and ρ = 0.99) in site × subject interactions and in site-level IQR and SD. The standard deviation of the interaction effects for each site had a median decrease of 7% after cell-type adjustment (Supplementary Figure 3).

### 3.8 Identification and distribution of hypervariable sites

The dataset had 9,573 CpG sites with coverage higher than 10x. We retained 4,786 sites, filtering at the 50th percentile of Hrel. Of the filtered-out sites, 4,217 had a mean methylation above 95% and 570 had below 5%.

We classified 302 (6.3%) sites as hypervariable as they exceeded a threshold of 0.25 IQR in the cell composition-adjusted model. Without adjustment, a larger proportion of sites (552, 12%) exceeded the same threshold.

**FIGURE 3:**
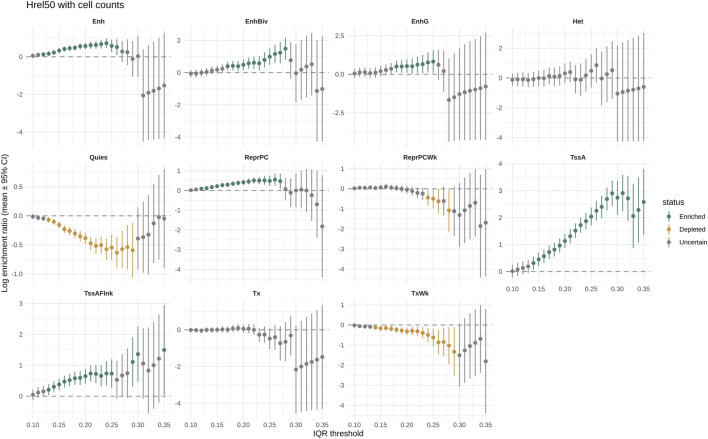
Enrichment in chromatin states for methylation variable sites selected at increasing IQR thresholds using posteriors from the complete model (Hrel50) with cell composition adjustment. For each chromatin state, a graph shows the log enrichment ratio (Y-axis) with 95% CI in the sites over the IQR threshold (X-axis). Chromatin states are from the Roadmap Epigenomics Project E030 sample lifted to the T2T-CHM13v2.0 reference.

**FIGURE 4:**
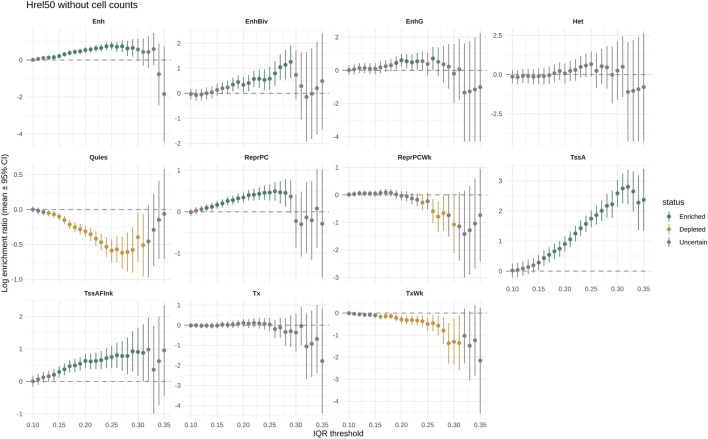
Enrichment in chromatin states for methylation variable sites selected at increasing IQR thresholds using posteriors from the complete model (Hrel50) without cell composition adjustment. For each chromatin state, a graph shows the log enrichment ratio (Y-axis) with 95% CI in the sites over the IQR threshold (X-axis). Chromatin states are from the Roadmap Epigenomics Project E030 sample lifted to the T2T-CHM13v2.0 reference.

Chromatin enrichments were generally stable from 0.15 IQR (Figures 3, 4). Excluding states with few observations (ZNF/Rpts and BivFlnk), we observed log enrichment in active promoters (TssA: 2.04; 95% CI: 1.71, 2.37), flanking promoters (TssAFlnk: 0.73; 95% CI: 0.27, 1.19), enhancers [Enh: 0.58 (0.25, 0.91)], bivalent enhancers [EnhBiv: 0.99 (0.48, 1.50)], genic enhancers [EnhG: 0.82 (0.08, 1.56)], and polycomb-repressed regions [ReprPC: 0.49 (0.23, 0.75)]. Depletion was observed for quiescent [Quies: −0.55 (−0.76, −0.33)], weakly transcribed [TxWk: −0.62 (−1.07, −0.18)], and weakly polycomb-repressed [ReprPCWk: −0.49 (−0.96, −0.02)] chromatin.

The enrichments were consistent across IQR thresholds (0.20, 0.25), in models with and without cell composition adjustment, and between brms and INLA implementations (Supplementary Figures 4, 5).

The 60th percentile datasets showed unstable results at an IQR of 0.15 but converged at 0.20 and 0.25. Enrichments near 0 were less stable; ReprPCWk was depleted only by two models without cell composition correction, and EnhBiv was not depleted in the 60th percentile datasets.

The tables with the chromatin enrichments are available in the publication-associated Dataverse.

The identification of hypervariable sites was not affected by coverage bias; that is, the mean coverage and IQR of the interaction effects had a small negative correlation (ρ = −0.09; Supplementary Figure 6).

The 302 hypervariable sites were unevenly distributed across genes, i.e., a higher proportion was present in *POMC* (22.0%, 39/177 sites), *IL-6* (16.7%, 13/78), *CRH* (14.8%, 12/81), and *OXTR* (10.5%, 29/277). Conversely, we observed lower proportions in *BDNF* (2.9%, 15/511), *NR3C1* (4.5%, 19/419), *FKBP5* (4.5%, 31/692), and *SLC6A4* (5.1%, 20/390).

Hypervariable sites also differed in annotations, being more likely to be in a CpG island (21% vs. 6%) and in a CDS (17.8% vs. 5.5%) and less likely to be in an intronic region (59.9% vs. 75.7%), with lower mean methylation (52.1% vs. 78.1%).

## 4 Discussion

Using INLA, we were able to efficiently fit different GLMMs to identify genomic sites with variable methylation. The results were comparable to those obtained with brms, but the runtimes were shorter by an order of magnitude.

The computation efficiency has two advantages. First, it allows running large-scale analyses that would be difficult, if not unfeasible, with an MCMC-based approach. Second, short running times are especially useful for exploratory data analysis and iterative prototyping, such as testing multiple predictors and systematic prior selection. As INLA supports simpler approximations (e.g., empirical Bayes), this advantage should extend to very large datasets.

Nevertheless, the concurrent analysis of a typical EWAS dataset remains unfeasible with any estimation method; 800,000 sites in 1,000 subjects would require estimating at least 800 million parameters for the interaction term. While a complete analysis is out of reach, this approach could be extended to larger genomic regions. For instance, this could be achieved by partitioning the genomic region of interest to fit separate models and then aggregating the results, potentially restricting the analysis to a subset of subjects. Our benchmark indicates that the approach could be computationally tractable with INLA as both the runtime and memory scaled almost linearly with the number of observations.

The site-specificity of the cell composition effects explains the high concordance between the adjusted and unadjusted models despite the better fit and discriminatory capability of the former. Accounting for cell composition remains necessary as the model similarity is likely to be specific for this dataset; other genomic regions could be more affected by cell composition. In addition, our cohort of healthy blood donors may be more homogeneous than other populations.

The genomic context of the identified hypervariable sites is compatible with localization in regulatory regions. The sites were enriched for active transcription start sites and bivalent enhancers and were more commonly present in CpG islands. Their increased proportion within CDS is compatible with a role in the regulation of mRNA isoform expression as DNA methylation modulates the inclusion of alternatively spliced exons (Shayevitch et al., 2018).

Hypervariable sites often had intermediate methylation rates. This is in agreement with previous studies showing that CpG sites with high inter-individual variability often have intermediate methylation levels. This variation was explained as changes in regulation within specific cell lineages, contrasting with changes in hypomethylated or hypermethylated sites typical of cell differentiation (Hachiya et al., 2017). The enrichment in hypervariable sites in *POMC* is consistent with its sensitivity to environmental and physiological cues and regulation through DNA methylation (Candler et al., 2019).

The limitations of this study should be noted. While our results are not specific to a single genomic location as we included sites from different chromosomes, the panel size is limited. Therefore, biological findings, such as chromatin enrichment or cell composition effects, may not extend to the full epigenome. The panel size could have also affected the estimation of computational costs, which may scale differently in larger datasets. Finally, by modeling the genomic positions as independent intercepts, we did not measure correlations between neighboring sites. These correlations could be estimated using spatial models (e.g., random walks), especially in the analysis of contiguous genomic regions.

In conclusion, using INLA, we were able to identify CpG sites with high inter-individual methylation variability. These sites had methylation rates and genomic annotations that were compatible with a regulatory role, making them potential candidates for future studies. INLA provided results that were comparable to those of MCMC-based methods at a fraction of the computational costs, making it applicable for large datasets.

## Data Availability

The datasets presented in this study and the code used to analyse and generate the data are publicly available at the University of Milan Dataverse https://doi.org/10.13130/RD_UNIMI/E5WJHP.

## Supplementary material

SUPPLEMENTARY FIGURE 1 Posterior distributions of the standard deviation for the group-level effect of TE taxon under different PC priors. The X-axis shows the SD, and the Y-axis shows the posterior density.

SUPPLEMENTARY FIGURE 2 Prior (dashed line) and posterior (solid line) distributions for standard deviation of the group-level effect of TE taxon under a subset of PC prior specifications. The X-axis shows the standard deviation. The Y-axis shows the density.

SUPPLEMENTARY FIGURE 3 Histogram showing the standard deviation reduction of the site × subject interaction effects between the model with and without cell composition adjustment.

SUPPLEMENTARY FIGURE 4 Enrichment in chromatin states for methylation variable sites, which were selected at increasing IQR thresholds, using posteriors from the model fitted with brms on the Hrel60 dataset. For each chromatin state, a graph shows the log enrichment ratio (Y-axis) with 95% CI in the sites over the IQR threshold (X-axis). Chromatin states are from the Roadmap Epigenomics Project E030 sample lifted to the T2T-CHM13v2.0 reference.

SUPPLEMENTARY FIGURE 5 Enrichment in chromatin states for methylation variable sites, which were selected at increasing IQR thresholds, using posteriors from the model fitted with INLA on the Hrel60 dataset. For each chromatin state, a graph shows the log enrichment ratio (Y-axis) with 95% CI in the sites over the IQR threshold (X-axis). Chromatin states are from the Roadmap Epigenomics Project E030 sample lifted to the T2T-CHM13v2.0 reference.

SUPPLEMENTARY FIGURE 6 Association (ρ = −0.09) between the site mean coverage (X-axis) and IQRs (Y-axis) of the interaction effects from the complete model with cell composition adjustment.
