# Supplementary figures and images for: Application of integrated nested Laplace approximation to identify hot spots of methylation heterogeneity in healthy individuals from the MAMELI cohort
Source: Front Genet. 2026 Apr 29;17:1787544. doi: 10.3389/fgene.2026.1787544 (PMC13167093; doi:10.3389/fgene.2026.1787544)

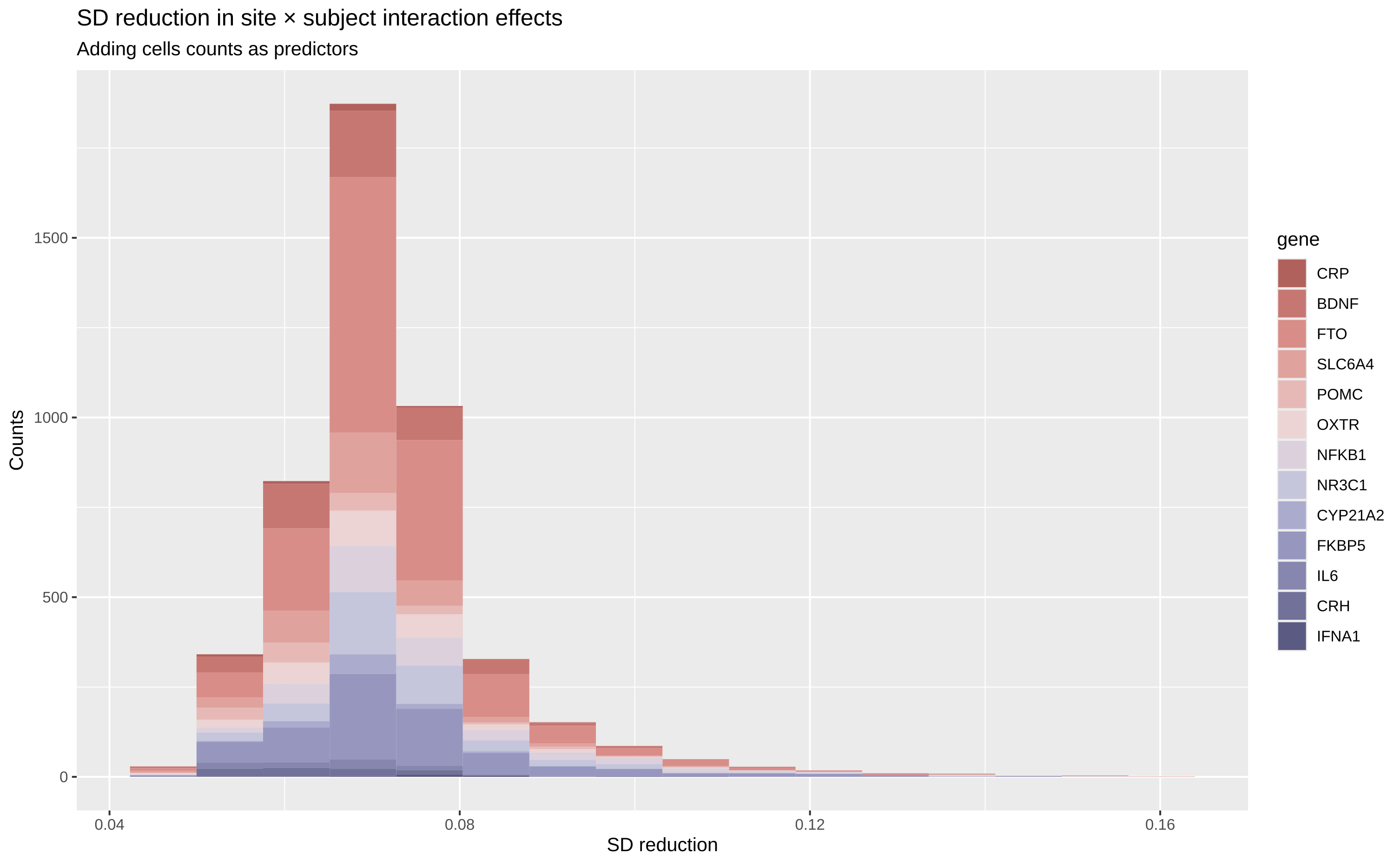

Supplement: Supplementary file 1 [file Image3.tiff]

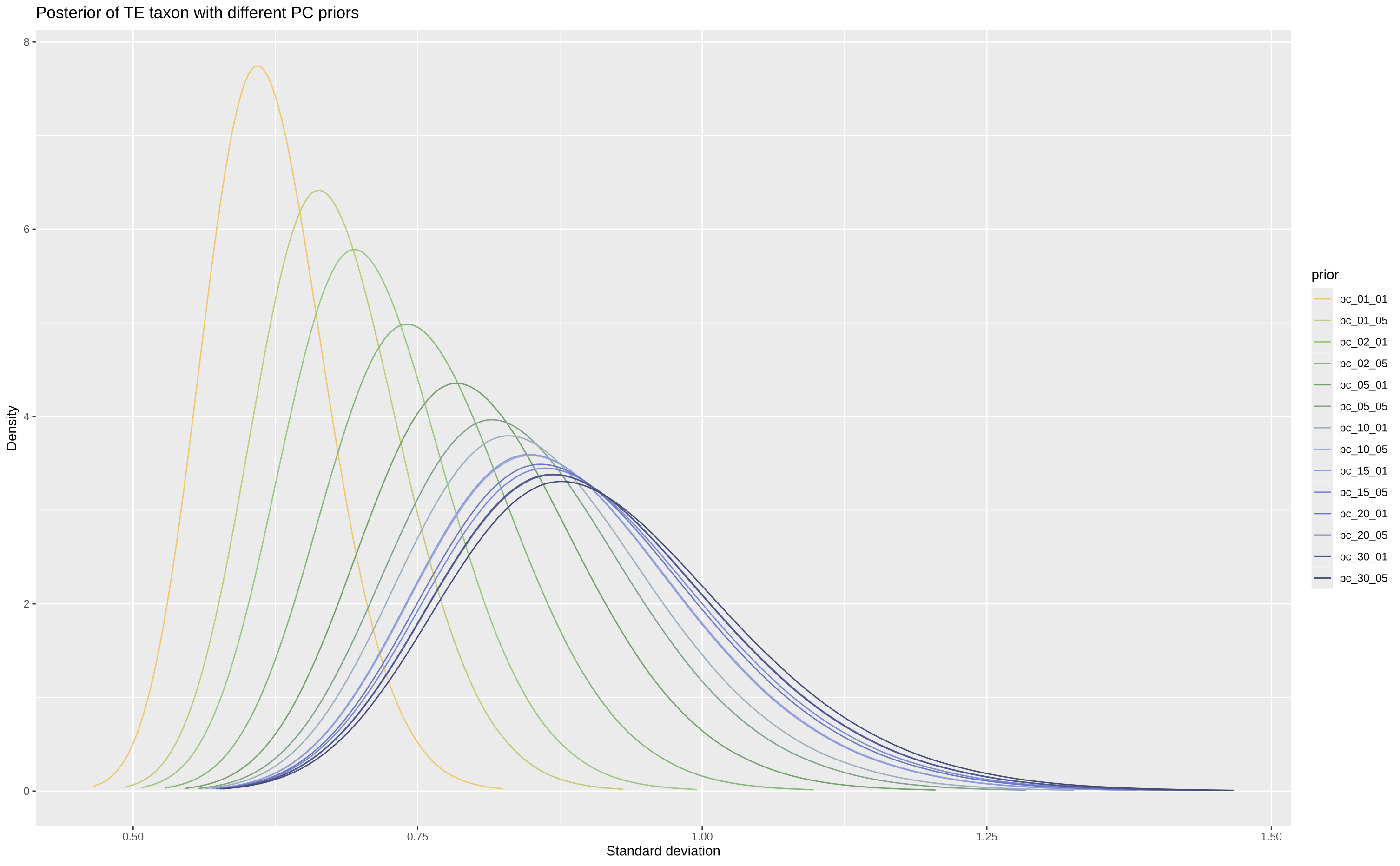

Supplement: Supplementary file 2 [file Image1.tiff]

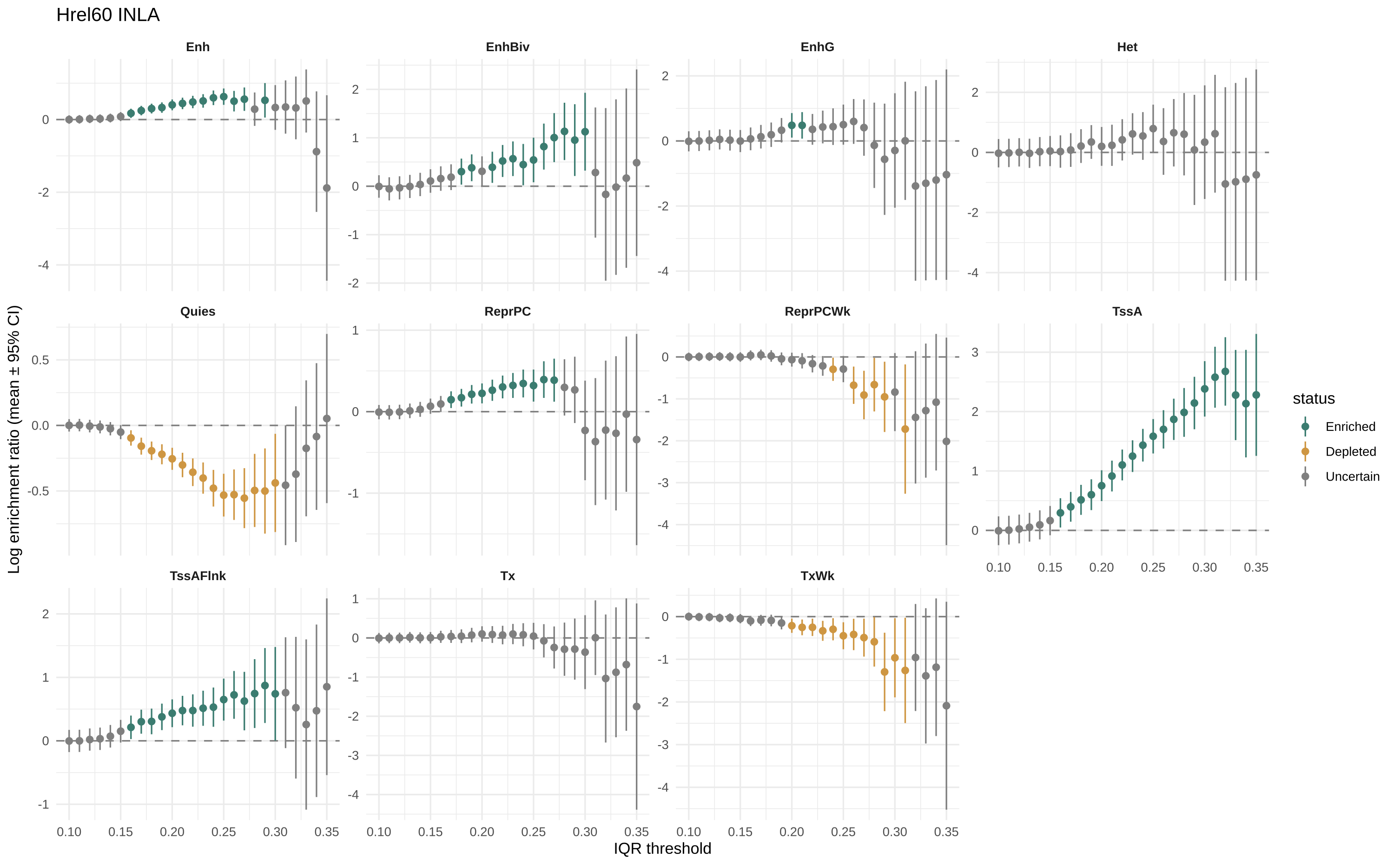

Supplement: Supplementary file 3 [file Image5.tiff]

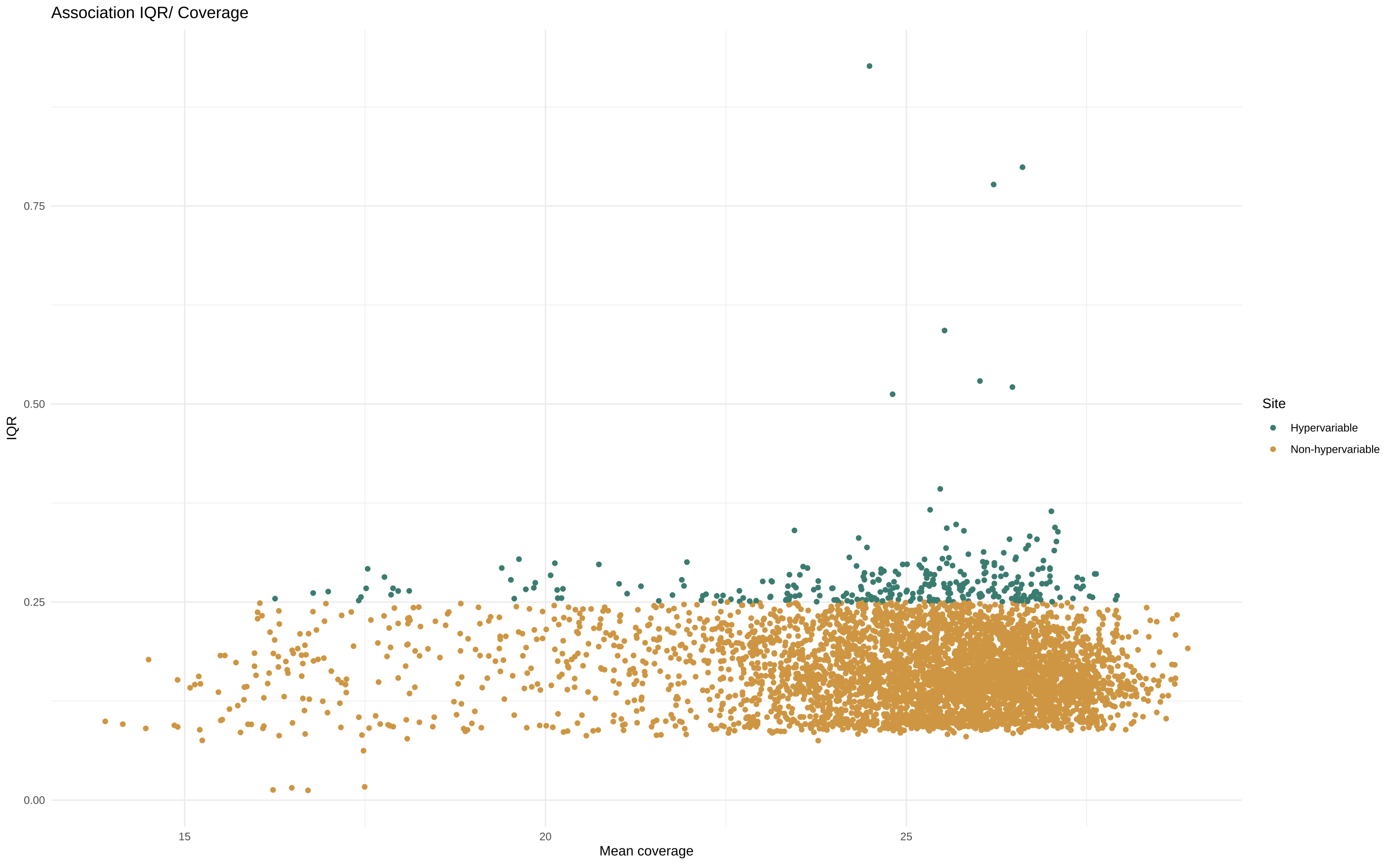

Supplement: Supplementary file 4 [file Image6.tiff]

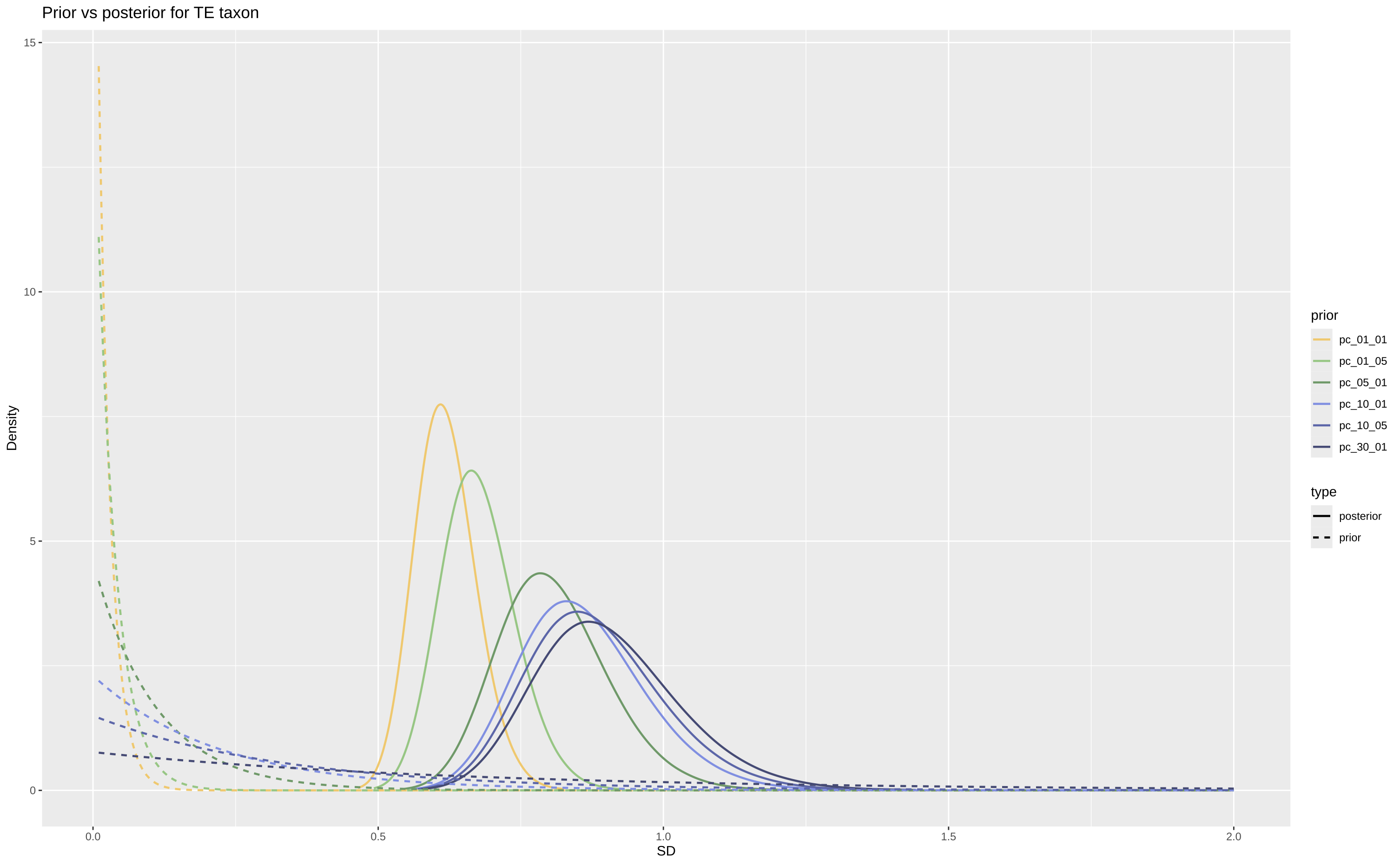

Supplement: Supplementary file 5 [file Image2.tiff]

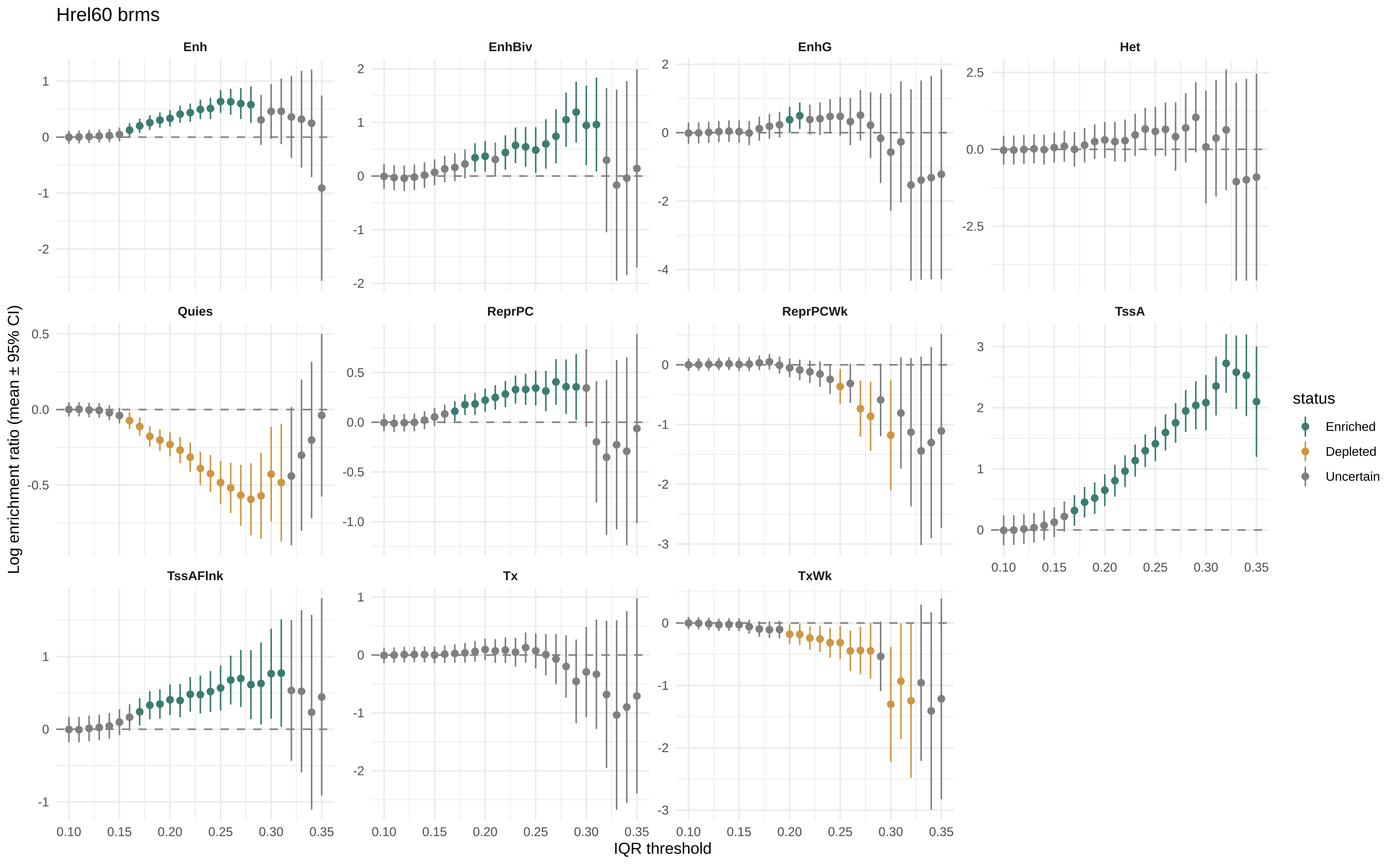

Supplement: Supplementary file 6 [file Image4.tiff]
